# Effect of Deep Brain Stimulation on Parkinson's Nonmotor Symptoms following Unilateral DBS: A Pilot Study

**DOI:** 10.4061/2011/507416

**Published:** 2011-12-19

**Authors:** Nelson Hwynn, Ihtsham Ul Haq, Irene A. Malaty, Andrew S. Resnick, Yunfeng Dai, Kelly D. Foote, Hubert H. Fernandez, Samuel S. Wu, Genko Oyama, Charles E. Jacobson, Sung K. Kim, Michael S. Okun

**Affiliations:** ^1^Center for Movement Disorders & Neurorestoration, College of Medicine, University of Florida, Gainesville, FL 32610, USA; ^2^Division of Neurology, Scripps Clinic Torrey Pines, La Jolla, CA 92037, USA; ^3^Department of Neurology, Wake Forest School of Medicine, Winston-Salem, North Carolina, USA; ^4^Department of Biostatistics, University of Florida, Gainesville, FL 32610, USA; ^5^Center for Neurological Restoration, Cleveland Clinic, Cleveland, OH, USA

## Abstract

Parkinson's disease (PD) management has traditionally focused largely on motor symptoms. Deep brain stimulation (DBS) of the subthalamic nucleus (STN) and globus pallidus internus (GPi) are effective treatments for motor symptoms. Nonmotor symptoms (NMSs) may also profoundly affect the quality of life. The purpose of this pilot study was to evaluate NMS changes pre- and post-DBS utilizing two recently developed questionnaires. *Methods*. NMS-Q (questionnaire) and NMS-S (scale) were administered to PD patients before/after unilateral DBS (STN/GPi targets). *Results*. Ten PD patients (9 STN implants, 1 GPi implant) were included. The three most frequent NMS symptoms identified utilizing NMS-Q in pre-surgical patients were gastrointestinal (100%), sleep (100%), and urinary (90%). NMS sleep subscore significantly decreased (−1.6 points ± 1.8, *P* = 0.03). The three most frequent NMS symptoms identified in pre-surgical patients using NMS-S were gastrointestinal (90%), mood (80%), and cardiovascular (80%). The largest mean decrease of NMS scores was seen in miscellaneous symptoms (pain, anosmia, weight change, and sweating) (−7 points ± 8.7), and cardiovascular/falls (−1.9, *P* = 0.02). *Conclusion*. Non-motor symptoms improved on two separate questionnaires following unilateral DBS for PD. Future studies are needed to confirm these findings and determine their clinical significance as well as to examine the strengths/weaknesses of each questionnaire/scale.

## 1. Introduction

The traditional approach to Parkinson's disease (PD) patients has focused heavily on addressing motor symptoms and on-off fluctuations. Deep brain stimulation (DBS) of the subthalamic nucleus (STN) and globus pallidus interna (GPi) are potentially effective treatments for these motor types of symptoms. Nonmotor symptoms (NMSs), however, are also common and debilitating and have been reported in up to 28% of PD cases. NMS have also been reported by some investigators as being more debilitating than motor symptoms [1]. Two scales that have recently been used to quantify nonmotor symptoms include the nonmotor Symptoms Questionnaire (NMS-Q) and the Non-motor Symptom Scale (NMS-S). Both scales were recently validated by Chaudhuri and colleagues [2–4]. The purpose of this pilot study was to evaluate NMS pre- and postoperatively in unilateral PD cases utilizing these scales to determine changes in overall NMS and to potentially guide a future study.

## 2. Methods

Consecutive PD patients undergoing initial unilateral DBS implantation at the University of Florida were screened. All subjects were between the ages of 18 to 85, had the diagnosis of idiopathic PD confirmed by a movement disorders specialist (utilizing UK Brain Bank criteria), and had a documented 30% or better improvement in motor performance following levodopa administration (as measured by the Unified Parkinson's Disease Rating Scale Part III). Subjects were excluded from DBS implantation and consequently from this study if they had significant and active psychiatric disease as determined by a semistructured clinical interview (SCID) or if neuropsychological testing revealed moderate to severe cognitive impairment (DRS score of <130) or dementia based on a neuropsychological profile indicating declines in multiple cognitive domains). The NMS-Q and NMS-S scales were used to follow nonmotor symptoms pre- and post-DBS. The NMS-Q was based on patient response with “yes” and “no” questions. The NMS-S was a clinician-based assessment questionnaire that both documents the presence of the non-motor symptoms as well as their frequency and duration. 

Utilizing the NMS-Q form, the subsets were divided (in an attempt to derive comparisons to the NMS-S) as follows: questions 1, 3, 4–7 were grouped under the *gastrointestinal* symptoms subset, questions 8-9 were grouped under the *urinary* symptoms subset, questions 2, 10-11 and 27-28 were grouped under the *miscellaneous* symptoms subset, questions 12-13 and 15 were grouped under the *cognition* symptoms subset, questions 16-17 were grouped under the *mood* symptoms subset, questions 18-19 were grouped under the *sexual dysfunction *symptoms subset, questions 20-21 were grouped under the *cardiovascular/falls* symptoms subset, questions 22–26 were grouped under the *sleep* symptoms subset, and questions 14, 29-30 were grouped under *perceptual* symptoms subset. A patient was counted as having the symptom if (s)he has a positive answer in at least one of the questions in the corresponding subset. 

Utilizing the NMS-S form, the subsets were divided as follows: questions 1-2 were grouped under the *cardiovascular/falls* symptom subset; questions 3–7 were grouped under the *sleep/fatigue* symptoms subset; questions 8–14 were grouped under the *mood* symptoms subset; questions 15–17 were grouped under the *perceptual* symptoms subset; questions 18–20 were grouped under the *cognition* symptoms subset; questions 21–23 were grouped under the *gastrointestinal* symptoms subset; questions 24–26 were grouped under the *urinary* symptoms subset; questions 27-28 were grouped under the *sexual dysfunction* symptoms subset; questions 29–32 were grouped under the *miscellaneous* symptoms subset.

The primary analysis included the changes observed at baseline versus 6 months on the total NMS-Q and NMS-S scales. As secondary outcomes we examined subscores; however, sample sizes limited the overall interpretation.

## 3. Results

Ten PD patients (9 STN implants, 1 GPi implant) were included in this pilot study. All patients were screened and consented for research participation in the Movement Disorders Center database (INFORM-PD). The mean age was 66.1 years ±7.8, and the mean disease duration was 9.9 years ±3. The average time from preoperative to postoperative testing was 12.1 months ±7.3 (range 1–25 months). The average change of UPDRS motor score was −6.4 points ±13.1.

The primary analysis for the NMS-Q revealed that the total score for NMS symptoms (total possible score 30 points) improved by an average of −3.7 points ±4.3 (*P* = 0.03) (Table 1). Secondary analyses of the sample were then performed. The three most prevalent NMS symptoms identified in pre-surgical patients were gastrointestinal (100%), sleep (100%), and urinary symptoms (90%). Postoperatively, the three most prevalent NMS symptoms were sleep (90%), urinary (90%), and cognition (60%) subsets. The largest mean decrease of NMS scores were sleep (−1.6 points ±1.8, *P* = 0.03), gastrointestinal (−0.8 points ±1.1, *P* = 0.09), and cardiovascular/falls (−0.5 ± 1.0, *P* = 0.23) subsets. Only the sleep subscore in this small sample size reached statistical significance. Some other NMS categories also improved (but did not reach statistical significance) postoperatively including sexual dysfunction (−0.22 points ±0.83), mood (−0.2 points ±0.8), and miscellaneous (unexplained pain, excessive sweating, leg swelling) items (−0.5 points ±1.8). Miscellaneous symptom scores increased (50% to 60%), while urinary scores were unchanged (90%).

Using the NMS-S (total possible score 384 points), the primary analysis revealed that the NMS scores improved by an average of −38.5 points ±30.9 (*P* = 0.01). A secondary analysis then revealed that three most prevalent NMS symptoms identified in pre-surgical patients were gastrointestinal (90%), mood (80%), and cardiovascular (80%) symptoms. Postoperatively, the most prevalent NMS symptoms were urinary (80%), mood (70%), sexual (60%), and gastrointestinal (60%) symptoms. The largest mean decrease of NMS scores was in the categories of mood (−9.3 points ±13.8), sleep (−9 points ±13.3), and miscellaneous (−7 points ±8.7). Only miscellaneous symptoms (pain, anosmia, weight change, and sweating) (−7 points ±8.7), and cardiovascular/falls (−1.9, *P* = 0.02) reached statistical significance (Tables 1 and 2). 

Reports of sexual symptoms increased slightly from 55.6% preoperatively to 60% postoperatively.

## 4. Discussion

Both scales we administered revealed an improvement in non-motor symptoms following unilateral DBS, although the sample size limited our understanding of specific symptoms that may change. Sleep was, however, one feature that emerged as potentially important. Sleep impairment has been reported recently by several prepost-DBS studies, and this may be supported by our data [5]. Changes revealed in cardiovascular status, autonomic features, or falling may also be reported by some patients undergoing DBS; however, the small sample size of this study make interpretation difficult. 

Nonmotor symptoms are common in PD patients and can be important in determining overall quality of life [6]. The effects of DBS on NMS symptoms in advanced PD cohorts have not been entirely clarified [7]. In a study by Witjas and colleagues, 40 PD patients implanted with bilateral STN DBS experienced significant benefits with sensory/painful fluctuations, dysautonomia (excessive sweating), and cognitive fluctuations [8]. In another study by Zibetti and colleagues, sleep and constipation were the only symptoms that improved following bilateral STN DBS surgery in 36 PD patients [9]. Our pilot data, although derived from a small number of patients, utilized two recently validated NMS questionnaires and did point to improvements in NMS and particularly improvements in sleep. 

Within our cohort, sleep, gastrointestinal, and cardiovascular/falls improved the most when using the NMS-Q while mood, sleep, and miscellaneous symptoms improved when employing the NMS-S. A possible explanation for the differences in the two scales may have been the number of symptoms attributed to each subscore. In the NMS-Q there were 2 questions that comprised the mood subset, while in the NMS-S, there were 7. In another example, the NMS-Q gastrointestinal subset was composed of 9 questions, while, in the NMS-S scale, it was only 3. Finally, mood symptoms were more commonly reported in the NMS-S, but not in the NMS-Q. Devoting more questions to a particular symptom could increase the sensitivity of any scale for a particular area, and this may have biased our dataset. While the changes in overall score were close between the two scales (~10% improvement), one might hypothesize for future investigations that greater information may be derived from the NMS-S (when compared to the NMS-Q) as it queries both frequency and severity of symptoms. 

The major limitation of this pilot study was the sample size. Our analysis yielded results which reached significance when employing the total scores of the scales (NMS-Q, *P* = 0.03; NMS-S, *P* = 0.01) but yielded less significant and impressive results when parceling subitems. Further, it is not clear what degree of change in these NMS questionnaires will prove clinically significant. We emphasize that there was a small sample size used in this pilot study, therefore, results should be considered preliminary and speculative. Future studies should focus on both STN and GPi (our sample was small for GPi) and should include both unilateral and bilateral cases preferably with a larger sample size and longer clinical followup. Based on the preliminary data, there is reason to believe that NMS have the potential to change pre- and post-unilateral DBS, and better understanding NMS will help us to achieve greater benefits and more realistic expectations from our DBS patients.

## Figures and Tables

**Table 1:** Changes in nonmotor symptom (NMS) score from questionnaire. The left-sided group shows percent of subjects who had a positive answer in at least one of the questions in the corresponding subset. The right-sided group shows changes in NMS score as represented by the difference in total score. Improvements of NMS scores prepost-deep brain stimulation surgery were significant. Also, sleep improved prepost-DBS (*P* = 0.03).

NMS	Frequency of NMS reported			NMS Total point		
	(% present)			difference		
	Pre-DBS	Post-DBS	*P* Value*	Mean	SD	*P* Value**
NMS-Q	—	—	—	−3.70	4.27	**0.03**
Gastrointestinal	100.0%	50.0%	NA	−0.80	1.14	0.09
Urinary	90.0%	90.0%	1.00	0.00	0.67	1.00
Miscellaneous	50.0%	60.0%	0.66	−0.50	1.84	0.45
Perceptual	30.0%	30.0%	1.00	0.20	0.63	0.63
Attention/memory	70.0%	60.0%	0.56	−0.10	0.62	0.81
Mood	60.0%	50.0%	0.56	−0.20	0.79	0.75
Sexual	55.6%	50.0%	1.00	−0.22	0.83	0.75
Cardiovascular/falls	80.0%	50.0%	0.18	−0.50	0.97	0.23
Sleep	100.0%	90.0%	NA	−1.60	1.84	**0.03**

*: *P*-values based on McNemar's test.

**: *P*-values based on Wilcoxon signed rank sum test.

**Table 2:** Changes in nonmotor symptoms (NMS) scale. The left-sided group shows prepost-DBS change in prevalence of NMS symptoms. The right-sided group shows changes in NMS score as a difference in total score. Improvements of NMS scores pre-post deep brain stimulation surgery were significant (*P* = 0.01). Mean scores in miscellaneous symptoms (*P* = 0.03) as well as cardiovascular/falls (*P* = 0.02) symptoms had significant improvement. Prevalence of CV/falls was also significantly different between pre- and post-DBS.

NMS	Frequency of NMS reported			NMS total point		
	(% present)			difference		
	Pre-DBS	Post-DBS	*P* value*	Mean	SD	*P* value**
NMS-S	—	—	—	−38.50	30.94	**0.01**
Gastrointestinal	90.0%	60.0%	0.18	−0.30	7.33	0.93
Urinary	70.0%	80.0%	0.56	−3.30	6.11	0.14
Miscellaneous	70.0%	40.0%	0.08	−7.00	8.69	**0.03**
Perceptual	20.0%	20.0%	1.00	0.00	1.25	1.00
Attention/memory	70.0%	60.0%	0.56	−3.50	8.20	0.30
Mood/cognition	80.0%	70.0%	0.56	−9.30	13.78	0.09
Sexual	55.6%	60.0%	1.00	−4.78	7.29	0.13
Cardiovascular/falls	80.0%	30.0%	**0.03**	−1.90	2.08	**0.02**
Sleep/fatigue	100.0%	100.0%	NA	−8.95	13.28	0.13

*: *P*-values based on McNemar's test.

**: *P*-values based on Wilcoxon signed rank sum test.

## References

[1] Lim SY, Lang AE (2010). The nonmotor symptoms of Parkinson’s disease—an overview. *Movement Disorders*.

[2] Chaudhuri KR, Martinez-Martin P, Schapira AHV (2006). International multicenter pilot study of the first comprehensive self-completed nonmotor symptoms questionnaire for Parkinson’s disease: the NMSQuest study. *Movement Disorders*.

[3] Chaudhuri KR, Martinez-Martin P, Brown RG (2007). The metric properties of a novel non-motor symptoms scale for Parkinson’s disease: results from an international pilot study. *Movement Disorders*.

[4] Chaudhuri KR, Martinez-Martin P (2008). Quantitation of non-motor symptoms in Parkinson’s disease. *European Journal of Neurology*.

[5] Lyons KE, Pahwa R (2006). Effects of bilateral subthalamic nucleus stimulation on sleep, daytime sleepiness, and early morning dystonia in patients with Parkinson disease. *Journal of Neurosurgery*.

[6] Barone P, Antonini A, Colosimo C (2009). The PRIAMO study: a multicenter assessment of nonmotor symptoms and their impact on quality of life in Parkinson’s disease. *Movement Disorders*.

[7] Sevillano-García MD, Manso-Calderón R (2010). Nonmotor symptoms in Parkinson’s disease and deep brain stimulation. *Revista de Neurologia*.

[8] Witjas T, Kaphan E, Azulay JP (2007). Effects of chronic subthalamic stimulation on nonmotor fluctuations in Parkinson’s disease. *Movement Disorders*.

[9] Zibetti M, Torre E, Cinquepalmi A (2007). Motor and nonmotor symptom follow-up in Parkinsonian patients after deep brain stimulation of the subthalamic nucleus. *European Neurology*.

